# Avatrombopag in heavily pretreated chronic immune thrombocytopenia: a single center experience from Oman

**DOI:** 10.3389/fmed.2026.1850341

**Published:** 2026-07-30

**Authors:** Nashwa Al-Sharji, Bushra Salman, Shireen Al-Zadjali, Mohammed Al-Huneini

**Affiliations:** 1 Department of Pharmacy, Sultan Qaboos University Hospital, University Medical City, Muscat, Oman; 2 Department of Pharmacy, National Hematology and Bone Marrow Transplant Center, University Medical City, Muscat, Oman; 3 Department of Adult Hematology, National Hematology and Bone Marrow Transplant Center, University Medical City, Muscat, Oman

**Keywords:** avatrombopag, immune thrombocytopenia, steroid-sparing effect, thrombopoietin receptor agonists, thrombopoietin-receptor-agonist

## Abstract

**Background:**

Chronic immune thrombocytopenia (ITP) is characterized by persistent thrombocytopenia and bleeding risk, and a subset of patients remains refractory or relapses despite multiple lines of therapy. Avatrombopag is an oral thrombopoietin receptor agonist approved for adults with chronic ITP with insufficient response to prior treatment. We descrixbe a real-world single-center experience with avatrombopag in Oman.

**Methods:**

We conducted a retrospective case series of consecutive adults with chronic primary ITP treated with avatrombopag at a tertiary care center in Muscat, Oman. Patients initiated avatrombopag between September 2023 and June 2025 and were followed through October 2025. Platelet counts, treatment patterns, transfusion requirements, corticosteroid use, rescue therapies, and adverse drug reactions (ADRs) were abstracted from electronic medical records.

**Results:**

Twelve heavily pretreated patients were included, with a median baseline platelet count of 14.5 × 10^9^/L (IQR 4.8–28.8). Eleven patients (91.7%) started avatrombopag at 20 mg once daily, and one patient (8.3%) started at 20 mg on alternate days; 66.7% required subsequent dose adjustment. Overall, 75.0% achieved platelet counts ≥ 50 × 10^9^/L at least once during follow-up, with a median platelet count of 160 × 10^9^/L at month 3 and 98 × 10^9^/L at the most recent assessment. Transfusion burden improved in 60% of patients with high baseline transfusion requirements. Steroid-sparing was observed in 83.3, and 66.7% were steroid-free during follow-up. ADRs occurred in 33.3% (primarily body pain and rash); no hepatotoxicity or treatment discontinuation due to ADRs was documented.

**Conclusion:**

In chronic, heavily pretreated ITP, avatrombopag was associated with clinically meaningful platelet responses, substantial steroid-sparing, and an acceptable tolerability profile.

## 1 Background

Immune thrombocytopenia (ITP) is an immune-mediated hematologic disorder characterized by isolated thrombocytopenia and variable bleeding risk (1). Although ITP is considered a rare disease, it represents a sustained clinical and health system burden due to relapse-prone disease biology, the need for repeated monitoring, and treatment-related toxicity (2). In adults, the incidence of primary ITP has been estimated at approximately 3.3 per 100,000 per year, with a prevalence of about 9.5 per 100,000; however, these estimates vary by country and case definitions (3, 4). Alongside the risk of life-threatening bleeding, patient-reported outcomes consistently demonstrate substantial impairment in health-related quality of life (HRQoL) among patients with ITP, which impacts daily functioning and productivity (5).

Current ITP management is individualized and aims to prevent clinically significant bleeding while minimizing treatment-related side effects. According to the American Society of Hematology (ASH), first-line treatment of adult ITP includes corticosteroids, intravenous immunoglobulin (IVIG), and intravenous anti-D immunoglobulin (2, 6–8). This achieves a response in 75% of patients, but 30–40% may develop corticosteroid resistance or intolerance (2). For patients who are refractory to first-line treatments, second-line options typically include thrombopoietin receptor agonists (TPO-RAs), rituximab, and splenectomy, with fostamatinib and other agents utilized depending on prior response, comorbidities, access, and patient preference (2, 6–8).

Despite therapeutic advances, a significant proportion of patients with ITP develop persistent disease activity with inadequate platelet recovery and/or ongoing bleeding. Contemporary reviews suggest that approximately 5%–30% of patients may continue to experience low platelet counts and/or ongoing bleeding despite available therapies (9). This subgroup is associated with a high risk of morbidity due to bleeding, infection due to cumulative immunosuppression, and intensive health care utilization (10).

TPO-RAs have become a cornerstone of second-line management of ITP by augmenting platelet production and enabling corticosteroid-sparing treatment strategies. Avatrombopag is an orally administered TPO-RA approved for thrombocytopenia in adults with chronic ITP who have had an insufficient response to a previous treatment (11). In contrast to eltrombopag, avatrombopag does not require dietary restrictions and has been characterized as having minimal clinically relevant food-chelation limitations, which may improve adherence in real-world settings (12). In the pivotal phase 3 randomized study, avatrombopag was superior to placebo in increasing and maintaining platelet counts over the treatment period, with early platelet responses observed and a tolerable safety profile (13). Subsequent analyses of the phase further supported the durability of platelet responses in patients receiving avatrombopag (14).

Given ongoing gaps in region-specific real-world evidence, additional data are needed to characterize the effectiveness and safety of avatrombopag in routine practice across diverse patient populations, including those with heavily pretreated or refractory disease features. This retrospective case series from Oman evaluated the real-world effectiveness and safety outcomes of avatrombopag in adults with chronic ITP treated in a tertiary care setting.

## 2 Materials and methods

The study was approved by the Ethics and Research Committee, Sultan Qaboos University Hospital, Oman (Ref No. SQU-EC/041/2025, MREC#3575). Given the retrospective design and use of de-identified routine-care data, the requirement for informed consent was waived in accordance with local regulations and institutional policy. Patient confidentiality was maintained throughout data extraction, analysis, and reporting. The manuscript was developed in alignment with STROBE principles for observational research

### 2.1 Study design and patients

A single-center retrospective case series was conducted in adult patients with chronic ITP who were treated with avatrombopag at the outpatient and inpatient units of Sultan Qaboos University Hospital and the National Hematology and Bone Marrow Transplant Center, University Medical City, Muscat, Oman. The study used routinely collected clinical data from the electronic medical records of patients who initiated avatrombopag from September 2023 to June 2025. The index date was defined as the date of initiation of avatrombopag for ITP. Each patient was followed from the index date until the earliest of avatrombopag discontinuation, last documented clinical contact, death, or end of the observation window (October 2025). To reduce selection bias, all consecutive eligible patients initiating avatrombopag during the study period were included.

Eligible patients were adults ( ≥ 18 years at the index data) with a clinician-documented diagnosis of primary ITP and chronic disease, defined as ITP duration > 12 months from diagnosis to avatrombopag initiation. Diagnosis was based on routine hematology assessment consistent with standard practice, including evaluation to exclude alternative causes of thrombocytopenia as documented in the medical record. Avatrombopag must have been initiated for treatment of ITP due to insufficient response, relapses, intolerance, or treatment-burden considerations with prior therapies. Eligible patients were required to have available platelet count data and clinical follow-up for at least 3–6 months after initiation of avatrombopag. Patients were excluded if they had non-immune or secondary thrombocytopenia that did not meet the diagnostic criteria for ITP.

Avatrombopag dosing and dose adjustments were determined by the treating physician. The usual starting dose was 20 mg once daily, with subsequent adjustment according to platelet count response, bleeding status, concomitant rescue therapy, tolerability, and concern regarding excessive platelet rise. Dose escalation was considered when platelet counts remained below the clinically desired threshold despite treatment, whereas dose reduction, treatment interruption, or lower-frequency maintenance dosing was considered when platelet counts increased excessively or remained above the desired range.

### 2.2 Variables and endpoints

Data were extracted from the electronic medical records, discharge summaries, laboratory results, and pharmacy data. Baseline characteristics were defined using the most recent values within (e.g., 30 days) prior to avatrombopag initiation. Variables collected included demographics; ITP history (disease duration, prior therapies, number of prior treatment lines, and prior exposure to TPO-RAs); relevant comorbidities; concomitant medications; and bleeding events throughout follow-up. Baseline laboratory parameters included platelet count and, where available, hemoglobin, white blood cell count, and liver function tests. Follow-up platelet counts were captured at all routine clinical assessments; when analyses required anchoring to prespecified timepoints (e.g., weeks 2, 4, 8, and 12). For weeks 2 and 4 assessments, platelet counts within ± 7 days of the target date were used. For later assessments, platelet counts within ± 14 days of the target date were used. Safety outcomes included adverse events documented during avatrombopag exposure

The primary endpoints were the change in platelet count following initiation of avatrombopag and platelet response. Platelet response was defined as a platelet count ≥ 30 × 10^9^/L with at least a two-fold increase from baseline and no clinically relevant bleeding. Complete response was defined as a platelet count ≥ 100 × 10^9^/L with no clinically relevant bleeding. Durable response was defined according to ASH terminology as a platelet count ≥ 30 × 10^9^/L with at least a two-fold increase from baseline at 6 months (15). Additional endpoints included safety outcomes; use and dose of corticosteroids or other immunosuppressants; the proportion of patients with clinically documented overt bleeding episodes or a high transfusion burden; and the proportion of patients with a steroid-sparing effect, defined as either a ≥ 50% reduction in corticosteroid dose or complete discontinuation during follow-up. Bleeding outcomes were extracted from clinician documentation in the electronic medical records. Because standardized bleeding scores were not routinely recorded, formal retrospective grading was not performed. For descriptive analysis, bleeding manifestations were categorized as cutaneous bleeding manifestations (including bruising, ecchymosis, or petechiae) and clinically documented overt bleeding episodes (including mucosal or other non-cutaneous bleeding, where documented). Severe or major bleeding was defined pragmatically as bleeding associated with hospitalization, hemodynamic compromise, red-cell transfusion, invasive intervention, critical-site bleeding, or death. Platelet transfusion burden was calculated as the number of platelet transfusion episodes divided by the duration of the corresponding observation period in weeks. High transfusion burden was defined as requiring one or more platelet transfusion episodes per week. Low transfusion burden was defined as requiring less than one platelet transfusion episode per week.

### 2.3 Statistical analysis

Data were summarized using descriptive statistics. Continuous variables were reported as means ± standard deviation (SD) or medians (ranges), and categorical variables as counts and percentages.

## 3 Results

### 3.1 Baseline characteristics

The study included 12 adult patients with chronic ITP who received avatrombopag (Table 1). Two-thirds were female (66.7%, *n* = 8). The median age at diagnosis was 25 years (IQR 21.5–45). All patients received ≥ 2 prior ITP therapies; 41.7% had ≥ 4 prior lines. Corticosteroids were used in 66.7%, intravenous immunoglobulin (IVIG) in 58.3%, and rituximab in 50%. Eleven patients (91.7%) had a history of prior TPO-RA (romiplostim in 58.3% and eltrombopag in 91.7%). Splenectomy was performed in 25% of the cohort. Thrombosis was reported in one patient before avatrombopag initiation. At baseline, patients exhibited moderate cytopenias, with overall hepatic function preserved. Platelet counts were severely reduced, with a median of 14.5 × 10^9^/L (IQR 4.8–28.8).

**TABLE 1:** Baseline demographics and clinical characteristics of the patients before starting avatrombopag.

Variable	Patients (*n* = 12)
Age in years, median (IQR)	42.0 (22.5–50.5)
Age at diagnosis in years, median (IQR)	25 (21.5–45)
Female, no. (%)	8 (66.7%)
BMI in kg/m^2^, mean (SD)	31.0 (10.6)
Duration of ITP in years, median (IQR)	8.0 (2.0–11.0)
Prior therapies, no. (%)	
TPO-RA	11 (91.7%)
- Eltrombopag	10 (83.3%)
- Romiplostim	7 (58.3%)
Corticosteroids	8 (66.7%)
IVIG	7 (58.3%)
Rituximab	6 (50.0%)
Prior line of therapies, no. (%)	
2	2 (16.7%)
3	5 (41.7%)
≥ 4	5 (41.7%)
Splenectomy, no. (%)	3 (25.0%)
History of thrombosis, no. (%)	1 (8.3%)
Laboratory parameters, median (IQR)	
Platelet count (×10^9^/L)	14.5 (4.8–28.8)
Hemoglobin in g/dL	10.40 (9.9–11.8)
Serum bilirubin in μmol/L	8.0 (6.8–11.5)
ALT in U/L	16.5 (13.0–24.3)
AST in U/L	18.5 (14.0–26.8)
History of platelet transfusion, median (IQR)	11.0 (0.0–17.0)

ALT, Alanine transaminase; AST, aspartate transaminase; ITP, Immune thrombocytopenia; IVIG, intravenous immunoglobulin.

### 3.2 Dosing and treatment patterns

Eleven patients started avatrombopag at 20 mg daily, while the remaining patient started avatrombopag at 20 mg as an individualized cautious starting regimen selected by the treating physician. Eight patients (66.7%) required at least one dose increase. Most patients who had adjustments required only one change (62.5%, *n* = 5). One patient needed three adjustments (12.5%), and two patients needed six adjustments (25%). At the last follow-up, four patients (33.3%) remained on 20 mg daily, one patient received 20 mg twice per week, and one patient was on 20 mg on alternate days. The other six patients were maintained on 40 mg daily. The median avatrombopag exposure at data cutoff was 10.5 (3.3–15.5) months, while the median overall follow-up was 10.5 months (IQR 3.3–15.5; range 1.0–25.0). During treatment, rescue IVIG was used in five patients (41.7%) for physician-determined clinical indications, including bleeding manifestations and the need for rapid platelet support. Two patients (16.7%) received concurrent romiplostim during avatrombopag treatment, reflecting individualized management in heavily pretreated patients with refractory disease (Table 2).

**TABLE 2:** Treatment patterns of avatrombopag and outcomes.

Variable	Patients (*n* = 12)
Starting dose, no. (%)	
20 mg/day	11 (91.7%)
20 mg on alternate days	1 (8.3%)
Avatrombopag exposure at data cutoff in months, median (IQR)	10.5 (3.3–15.5)
Follow-up in months, median (IQR)	10.5 (3.3–15.5)
Dose adjustment, no. (%)a	
1	5 (41.7%)
3	1 (8.3%)
6	2 (16.7%)
Dose at last follow-up, no. (%)	
20 mg/day	4 (33.3%)
20 mg on alternate days	1 (8.3%)
20 mg 2 days per week	1 (8.3%)
40 mg/day	6 (50.0%)
Concurrent treatment, no. (%)	
IVIG	5 (41.7%)
Romiplostim	2 (16.7%)

^a^Dose adjustment was performed according to platelet count response, bleeding status, concomitant rescue therapy, tolerability, and treating physician judgment. The objective was maintenance of a clinically safe platelet count using the lowest effective avatrombopag dose, rather than platelet count normalization. IVIG, intravenous immunoglobulin

### 3.3 Outcomes

**FIGURE 1:**
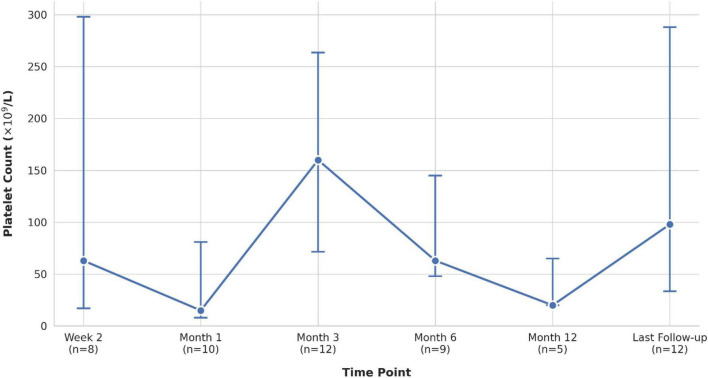
Median and interquartile ranges of platelet counts over time.

At baseline, platelet counts were low, with a median of 14.5 × 10^9^/L (IQR 4.8–28.8); 11 patients (91.7%) had baseline platelet counts < 50 × 10^9^/L, and 9 (75.0%) were < 30 × 10^9^/L. Median platelet counts increased as early as week 2, with a median of 63.0 × 10^9^/L (IQR 32.0–94.8). Platelet responses ranged from minimal increases (3 × 10^9^/L) to marked thrombocytosis (425 × 10^9^/L and 767 × 10^9^/L). At 1-month post-treatment, the median platelet counts were 15 × 10^9^/L (IQR 8–81), with a mean (SD) of 76.5 × 10^9^/L (123.2). By month 3, platelet responses showed improvement. The median platelet count increased to 160 × 10^9^/L (IQR 71.5–263.5), with a mean (SD) of 174.4 × 10^9^/L (135.97). Nine patients had platelet data at month 6. The median platelet count was 63 × 10^9^/L (IQR 48–145), with a mean (SD) of 106.3 × 10^9^/L (97.7). The median platelet count at the most recent assessment was 98 × 10^9^/L (IQR 33.5–288) (Figure 1).

Using standardized platelet-count response criteria, 8 of 11 evaluable patients (72.7%) achieved platelet response ( ≥ 30 × 10^9^/L with at least a two-fold increase from baseline). Complete response was also achieved by 8 of 11 patients (72.7%). Among responders, the median time to first platelet count ≥ 30 × 10^9^/L was 2.0 months (range 0.5–6.0). Durable response at month 6 was observed in 4 of 9 patients with available month 6 data (44.4%). Sustained achievement of platelet count ≥ 50 × 10^9^/L at two or more post-baseline assessments was observed in 7 of 11 evaluable patients (63.6%).

Cutaneous bleeding manifestations, including bruising or ecchymosis, were documented in 9 patients (75.0%) before avatrombopag initiation and in 4 patients (33.3%) during follow-up. Clinically documented overt bleeding episodes were recorded in 10 patients (83.3%) before avatrombopag initiation and in 4 patients (33.3%) during follow-up. No severe major bleeding events were documented during avatrombopag exposure.

Compared with baseline, 71% (5/7) of patients with low baseline transfusion needs remained in the low category post-avatrombopag, while 60% (3/5) of those with high baseline transfusion burden improved to the low category after treatment (Table 3). Two patients (16.7%) received concurrent romiplostim during avatrombopag exposure. In both patients, romiplostim was introduced because of inadequate or suboptimal platelet response and was therefore considered rescue/concurrent TPO-RA therapy rather than evidence of avatrombopag monotherapy response.

**TABLE 3:** Clinical and laboratory outcomes of avatrombopag.

Variable	Baseline (*n* = 12)	Last follow-up (*n* = 12)
Laboratory parameters, median (IQR)		
Hemoglobin in g/dL	10.40 (9.9–11.8)	10.9 (9.9–12.3)
Serum bilirubin in μmol/L	8.0 (6.8–11.5)	7.0 (4.5–10.5)
ALT in U/L	16.5 (13.0–24.3)	17.0 (13.0–34.5)
AST in U/L	18.5 (14.0–26.8)	20.5 (15.5–29.5)
Platelet response, no. (%)		
Any platelet response: ≥ 30 × 10^9^/L and ≥ 2-fold baseline increase (*n* = 11)	–	8 (72.7%)
Any complete response: ≥ 100 × 10^9^/L (n = 11)	–	8 (72.7%)
Durable response at month 6 (*n* = 9)	–	4 (44.4%)
Sustained ≥ 50 × 10^9^/L at ≥ 2 follow-up time points (*n* = 11)	–	7 (63.6%)
Platelet transfusion after treatment, median (IQR)	11.0 (0.0–17.0)	1.5 (0–18)
Transfusion burden, no (%)		
Low	7 (58.3%)	8 (66.7%)
High	5 (41.7%)	4 (33.3%)
Cutaneous bleeding manifestations, no. (%)	9 (75%)	4 (33.3%)
Clinically documented overt bleeding episodes, no. (%)	10 (83.3%)	4 (33.3%)
Steroids, no. (%)		
Steroid-free	3 (25.0%)	8 (66.7%)
Dose reduction	–	2 (16.7%)
Dose increase	–	2 (16.7%)
Steroid-sparing effect, no. (%)	–	10 (83.3%)
IVIG use, no. (%)	7 (58.3%)	5 (41.7%)

ALT, Alanine transaminase; AST, aspartate transaminase; ITP, Immune thrombocytopenia; IVIG, intravenous immunoglobulin.

At initiation of avatrombopag, only three patients (25%) were steroid-free. Following avatrombopag initiation, 66.7% (*n* = 8) of the cohort were steroid-free, including complete steroid discontinuation in four patients and ≥ 50% dose reduction in two patients. During avatrombopag therapy, 33.3% (*n* = 4) of patients experienced ADRs. Mild to moderate body pain and rash were the two most common toxicities reported, with one case of severe body pain. No hepatotoxicity, bleeding worsening, treatment discontinuation due to ADRs, or incident thromboembolic events were reported.

## 4 Discussion

In this retrospective single-center cohort from Oman, avatrombopag was used in a heavily pretreated chronic ITP population with long disease duration and frequent prior exposure to multiple therapies, including other TPO-RAs. Despite this refractory profile, most patients achieved clinically meaningful platelet improvement, with parallel reductions in bleeding manifestations, transfusion requirements, and corticosteroid use. These findings support avatrombopag as a practical option in routine care, particularly in patients who require repeated rescue therapy or in whom long-term steroid exposure remains a major source of morbidity.

The efficacy observed in our cohort is broadly consistent with the established avatrombopag literature, although cross-study comparison should be interpreted cautiously because our patients were more treatment-experienced and concomitant rescue therapy was allowed in real-world practice. In the pivotal phase 3 trial, avatrombopag achieved a median cumulative 12.4 weeks of platelet response versus 0.0 weeks with placebo, with a day 8 response rate of 65.6% versus 0.0% and a durable response rate of 34.4% versus 0.0% (13). In the subsequent *post hoc* analysis, response and complete response rates were 65.6%/37.5% by day 8, 84.4%/71.9 by day 28, and 87.5%/81.3% by month 6; 57.1% of patients on chronic corticosteroids reduced or discontinued steroids (14). In our series, platelet counts rose as early as 2 weeks, which is directionally similar, although durability is harder to estimate in a retrospective cohort where rescue IVIG and other concurrent interventions may be used pre-emptively.

The international signal has been broadly reproduced across populations. In Japanese adults, avatrombopag produced a day 8 response rate of 63.2 and a durable response rate of 42.1% (16); in Chinese adults, the corresponding figures were 72.9 and 43.8%, with a week 6 response rate of 77.1% versus 7.7% on placebo (17). In REAL-AVA 1.0 from the United States, 35% of baseline steroid users discontinued steroids, 57% of baseline immunosuppressant users discontinued immunosuppressants, and 66.8% of patients required no rescue medication during follow-up (18). Together, these data provide an external benchmark suggesting that the response profile observed in Oman is biologically plausible and consistent with established trial evidence, while also highlighting the value of contextual real-world series for characterizing outcomes in routine-care settings with distinct treatment pathways and resource constraints.

The steroid-sparing effect is one of the most clinically relevant findings of this study and warrants emphasis as a separate outcome. At baseline, many patients required ongoing corticosteroids, whereas during follow-up, the proportion who were steroid-free increased substantially, and most patients achieved some degree of steroid reduction or discontinuation. This is important because current guidelines increasingly discourage prolonged corticosteroid use and favor early transition to second-line therapy in patients who relapse, become steroid-dependent, or cannot maintain safe platelet counts without repeated steroid exposure (6, 8). The steroid-sparing signal in our cohort is also consistent with avatrombopag trial and program data showing corticosteroid reduction during treatment (14, 19), as well as real-world data showing decreased reliance on concomitant steroids after avatrombopag initiation (14, 19). In practice, this matters not only for toxicity reduction but also for lowering infection risk, metabolic complications, and healthcare utilization associated with chronic steroid use.

A balanced interpretation of avatrombopag also requires comparison with other TPO-RAs. Overall, avatrombopag, eltrombopag, and romiplostim all have established efficacy in chronic ITP, and there is no definitive head-to-head evidence proving large differences in effectiveness across all settings. Instead, treatment choice often depends on prior response, route of administration, safety considerations, adherence, and practical issues. Avatrombopag offers oral administration without the dietary polyvalent cation restrictions required with eltrombopag and does not carry the same boxed warning regarding hepatotoxicity, which may simplify routine use and monitoring in some patients. Romiplostim remains an effective alternative, particularly when weekly supervised dosing is preferred, but it requires subcutaneous administration. In a network meta-analysis, relative to other second-line agents in indirect comparison, avatrombopag improved durable response versus placebo and was associated with less any-grade bleeding than eltrombopag and romiplostim, with IRRs of 0.38 versus eltrombopag and 0.38 versus romiplostim, while no significant differences in overall adverse events were detected (20).

An additional practical observation from our cohort was the need for individualized dose modification during treatment. Although avatrombopag is commonly initiated at standard oral dosing, several patients in our series required tailored schedules, including alternate-day dosing, temporary step-down approaches, or twice-weekly administration, to maintain platelet control while limiting excessive platelet rise. This reflects an important real-world feature of TPO-RA use in chronic ITP: treatment success may depend not only on whether a patient responds, but also on how effectively dosing is titrated over time in response to platelet kinetics, bleeding risk, prior rescue therapy, and fluctuation in disease activity. In this respect, our experience suggests that avatrombopag can be flexibly individualized in routine practice, which may be particularly relevant in heavily pretreated patients with variable response trajectories.

Safety outcomes in our cohort were also broadly consistent with prior reports. Adverse drug reactions were mainly pain and rash, no hepatotoxicity was documented, and no patient discontinued treatment because of toxicity. This is in line with published clinical trials and real-world data indicating that avatrombopag is generally well tolerated (14, 19). At the same time, the occurrence of marked thrombocytosis in a small number of patients highlights the importance of dose titration and close platelet monitoring, especially early in treatment and in patients receiving concomitant rescue therapy. As with all TPO-RAs, thrombotic risk remains a relevant class consideration in ITP and is shaped by patient-specific factors such as age, comorbidity burden, prior thrombosis, and prothrombotic exposures (6). Although no overt thromboembolic signal emerged in this small cohort, careful baseline risk assessment and continued vigilance remain appropriate.

Several limitations should be acknowledged. This was a small retrospective single-center study, so precision is limited and selection bias is unavoidable. Treatment exposure and follow-up intensity were heterogeneous, and concomitant therapies, including rescue IVIG and occasional concurrent romiplostim, complicate causal attribution of response durability to avatrombopag alone. Bleeding outcomes were assessed descriptively rather than with standardized bleeding scores, and patient-reported outcomes were unavailable. Treatment exposure and follow-up intensity were heterogeneous, and concomitant therapies, including rescue IVIG, platelet transfusion, corticosteroids, and occasional overlapping romiplostim, limited causal attribution of platelet response durability to avatrombopag alone. Finally, in the absence of a comparator arm, formal conclusions regarding relative effectiveness versus other second-line strategies or other TPO-RAs cannot be made.

## 5 Conclusion

In this retrospective single-center Omani cohort of heavily pretreated chronic ITP, avatrombopag was associated with clinically meaningful platelet recovery in most patients, alongside a marked steroid-sparing effect and reductions in bleeding- and transfusion-related burden in those with higher baseline utilization, with an overall tolerability profile consistent with published trial and real-world experience and no evident hepatotoxicity or treatment discontinuations due to adverse events. While these findings support avatrombopag as a practical maintenance option for treatment-experienced ITP in routine care, interpretation is constrained by the small sample size, retrospective design, heterogeneous prior therapies and concomitant rescue treatments, and the lack of standardized bleeding and patient-reported outcomes, highlighting the need for larger multicenter regional studies with harmonized endpoints to define optimal sequencing, dosing, and long-term durability and safety.

## Data Availability

The raw data supporting the conclusions of this article will be made available by the authors, without undue reservation.
